# New Insights into Dose-Dependent Effects of Curcumin on ARPE-19 Cells

**DOI:** 10.3390/ijms232314771

**Published:** 2022-11-25

**Authors:** Giulia Carozza, Annamaria Tisi, Annamaria Capozzo, Benedetta Cinque, Aldo Giovannelli, Marco Feligioni, Vincenzo Flati, Rita Maccarone

**Affiliations:** 1 Department of Biotechnological and Applied Clinical Sciences, University of L’Aquila, 67100 L’Aquila, Italy; 2 Department of Life, Health & Environmental Sciences, University of L’Aquila, 67100 L’Aquila, Italy; 3 European Brain Research Institute, 00161 Rome, Italy; 4 Department of Neurorehabilitation Sciences, Casa di Cura Policlinico, 20144 Milano, Italy

**Keywords:** retinal pigment epithelium, ARPE-19, curcumin, autophagy, cell cycle, LC3BII, p62

## Abstract

Opposing dose-dependent effects of curcumin (Cur) have been documented in Retinal Pigment Epithelium (RPE); therefore, to shed the light on the mechanisms of action is crucial for ophthalmic applications. On this basis we explored new insights about the dose-dependent mechanisms triggered by Cur in human retinal pigment epithelial cells (ARPE-19). Three concentrations (0.01 mM; 0.05 mM; 0.1 mM) of Cur were tested, followed by morphological, molecular, and functional analysis of the cells. Cur 0.01 mM promotes a significant increase in cell proliferation, not affecting cell cycle progression and apoptosis; by contrast, Cur 0.05 mM and 0.1 mM block cellular proliferation and trigger S-phase cell cycle arrest without inducing apoptosis. The observation of *neuronal-like* morphological changes in Cur 0.05 mM and 0.1 mM were not associated with neuronal differentiation, as observed by the quantification of Neurofilament-200 and by the analysis of voltage-dependent currents by patch clamp. Evaluation of autophagic markers LC3BII and p62 revealed significant modulations, suggesting an important activation of autophagy in ARPE-19 cells treated with Cur 0.05 mM and Cur 0.1 mM; conversely, Cur 0.01 mM did not affect autophagy. Altogether, our findings show new dose-dependent mechanisms of action of Cur that suggest a wide therapeutic application in ocular diseases with different pathogenesis (i.e., proliferative vitreoretinopathy or Age-Related Macular Degeneration).

## 1. Introduction

Retinal Pigment Epithelium (RPE) is a monolayer of polarized and pigmented epithelial cells placed between the photoreceptor’s layer and the Bruch’s membrane (BM). Together with the BM and the choriocapillaris, RPE is one of the main components of the outer Blood-Retinal Barrier (BRB). Indeed, RPE contributes to the preservation of a controlled environment within the retina through the presence of tight junctions on its structure, which allow the correct maintenance of the visual function [1]. RPE plays several roles, including (a) the controlled transport of ions and nutrients from the choroid to the photoreceptors and vice-versa, (b) the regeneration of visual pigments during the visual cycle, particularly the re-isomerization of 11-*cis*-retinal which is not restored by photoreceptors (PRs), (c) the phagocytosis of PRs outer segments (POS) through the LC3-associated phagocytosis (LAP), and (d) the secretion of numerous growth factors (PDGF, PEDF, FGF, VEGF, and CNTF), which allows the proper functioning of the entire retina [2]. RPE dysfunction/degeneration is one of the first events involved in the pathogenesis of various proliferative or degenerative retinal diseases. Some examples include Proliferative Vitreoretinopathy (PVR), which is a common cause of surgical failure after the repair of rhegmatogenous retinal detachment [3], and Age-Related Macular Degeneration (AMD), the leading cause of blindness worldwide in the elderly [4]. In the past few years, many researchers have focused their studies on testing natural and biologically active molecules and their possible therapeutic application in retinal diseases. Among them, curcumin (Cur) is particularly important because of its versatility. Curcumin is the main curcuminoid extracted from the rhizome of *Curcuma longa*. It is a liposoluble polyphenol with low molecular weight, commonly used as a flavour agent [5]. Curcumin is considered a pleiotropic compound because of its ability to interact with many cellular components and influence a high number of cellular mechanisms [6]. Nevertheless, curcumin is known to exert opposite effects on different cell types, depending on the dose applied [7,8]. This was also demonstrated in RPE cells, in which concentrations up to 20 μM promoted (a) antioxidant effects through the activation of enzymatic and non-enzymatic molecules [9,10], (b) the down-regulation of Nuclear Factor kB (NF-kB) with a consequent decrease in the release of pro-inflammatory cytokines (IL-1β, IL-6) [9], (c) the modulation of Bax/Bcl-2 ratio, and (d) down-regulation of pro-apoptotic genes [11]. Conversely, concentrations above 20 μM induced proliferation block and cell cycle arrest followed by necrotic or apoptotic cell death [7,8]. Some evidence suggests that curcumin is also able to modulate autophagy, which is a self-degradative and conserved process [12]. In physiological conditions, autophagy allows the maintenance of cellular homeostasis through the degradation of misfolded protein and damaged organelles [13]. Specifically, in RPE cells, the autophagy process is involved in the renewal of photoreceptor outer segments (POS) and the regeneration of pigments during the visual cycle [14]. As mentioned above, both of these processes are performed by a non-canonical form of autophagy called LAP, in which autophagy cooperates with phagocytosis [15]. Maintenance of the correct level of autophagy activity is fundamental in the visual system; indeed, excessive activation or inhibition of this process contributes to the onset of pathological events in RPE. Particularly, during the pathogenesis of AMD, the impairment of autophagy leads to the accumulation of waste and non-degradable material called lipofuscin, inducing inflammation and oxidative stress burden, which accelerate the progression of the pathology [16]. A correct autophagy process is also implicated in the prevention of Epithelial to Mesenchymal transition (EMT) occurring during the pathogenesis of PVR [17,18]. In this scenario, RPE cells undergo EMT, de-differentiate into fibroblast-like cells, and acquire the ability to migrate, leading to the accumulation of extracellular matrix [3]. However, the molecular mechanisms involved in the pathogenesis of retinal dystrophies are still unclear. Angiogenesis, extracellular matrix integrity, isoprenoid-mediated responses, physiological or pathological autophagy, cell death induction, and retinal cell rescue are among the main mechanisms involved in retinal degenerative processes [19]. Previous studies have already addressed the capability of curcumin to induce the activation or inhibition of autophagy in different in vitro and in vivo models by different mechanisms [12]. It is known that autophagy impairment is involved in pathologies related to aging or neurodegeneration; thus, targeting this process is important for the development of therapeutic strategies. Curcumin modulation of autophagy was therefore studied in the central nervous system (CNS). On the one hand, it was shown that curcumin is able to activate autophagy in pathologies where this process is impaired; on the other, curcumin inhibits autophagy in those diseases with autophagy up-regulation [6,12]. These features support the versatility of curcumin and its possible therapeutic applications in a wide range of pathologies. For these reasons the deepening of the mechanisms through which curcumin acts on RPE cells may add the piece of knowledge necessary for the applicability of curcumin in ophthalmic diseases. In our study, we investigated the dose-dependent cellular and molecular mechanisms of curcumin in human retinal pigment epithelial cells (ARPE-19).

## 2. Results

### 2.1. Changes in Cell Viability and Cell Cycle Progression upon Curcumin Administration

**Figure 1:**
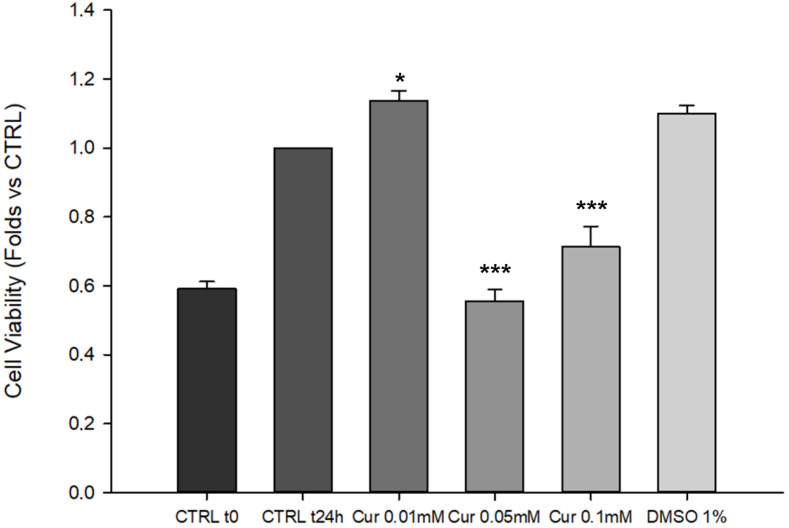
Cur effects on cell viability of ARPE-19 assessed by CCK-8 assay. Data are shown as mean ± SE. Statistical analysis was performed using one-way ANOVA test followed by the Holm-Sidak test. * *p* < 0.05; *** *p* < 0.001 versus CTRL t24 h (n = 3). The comparison between the treated groups and the baseline (CTRL t0) was carried out by calculating the mean differences.

To date, discordant data are reported on the outcomes induced by curcumin (Cur) supplementation on cell viability [7,20]. Thus, the first analysis of this study was the evaluation of cell viability in Cur-treated ARPE-19 cells by using the CCK-8 assay (Figure 1). As shown in Figure 1, 0.01 mM of Cur induced a significant increase in ARPE-19 cell proliferation compared to the CTRL t24 (*p* = 0.038). Notably, Cur 0.05 mM and 0.1 mM caused a significant decrease in cell viability compared to the CTRL at t24 h (*p* < 0.001). The CTRL t0 was used as a baseline in order to evaluate the differences in the proliferation rate of the treated groups compared to the starting point. The mean differences between Cur (0.05 mM and 0.1 mM)-treated cells and CTRL t0 were 0 and 0.1 ± 0.06 respectively, which suggest a proliferation arrest. The vehicle (DMSO 1%) did not exert any effects on cell proliferation, indicating that the proliferation arrest is induced exclusively by Cur (cell viability of DMSO 0.1% and 0.5% also did not alter cell proliferation, as reported in Supplementary Figure S1). 

This data suggests that curcumin influenced the proliferation of ARPE-19 cells in a dose-dependent manner. 

**Figure 2:**
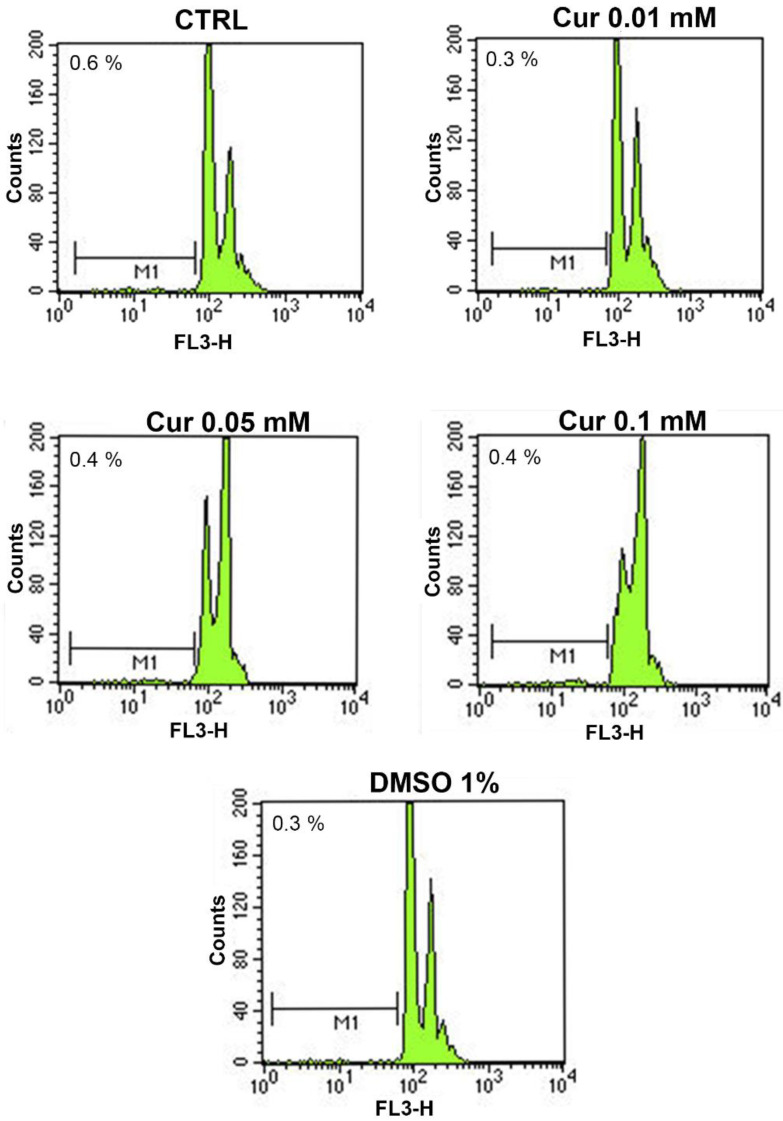
Profiles of apoptosis levels in ARPE-19 cells treated with curcumin (0.01 mM, 0.05 mM, and 0.1 mM) and DMSO 1% obtained by flow cytometry. Green peaks represent cell distribution. The representative profiles of all the vehicles are reported in the Figure S2.

Based on the cell viability assay, we wondered whether high Cur dosages induced a proliferation arrest or cell death. It is known that Cur can elicit different effects on several cellular processes such as cell death, inflammation, oxidative stress, angiogenesis, and autophagy, depending on the dose administered [6,8,9,12,21,22,23,24,25,26]. Hence, we used flow cytometry for the analysis in apoptosis levels. We found that Cur did not induce a relevant increase of apoptosis rate after a 24 h treatment in all the tested concentrations, compared to the control. Representative profiles are shown in Figure 2 and the mean ± SE of each experimental group was reported in Table 1.

**Figure 3:**
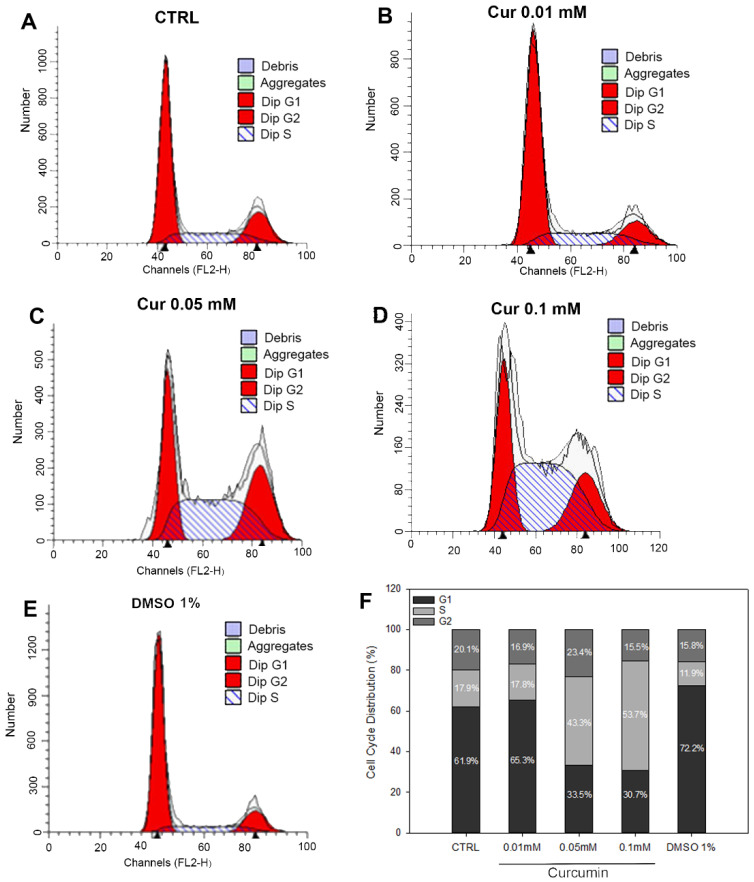
Cell cycle analysis obtained through flow cytometry. Representative profiles of cell cycle phases in (**A**) CTRL, (**B**) Cur 0.01 mM, (**C**) Cur 0.05 mM, (**D**) Cur 0.1 mM, (**E**) DMSO 1%. Debris and aggregates were not detected (**F**). Percentage distribution of Cur treated ARPE-19 cells within cell cycle phases. Representative profiles of cell cycle phases of all vehicles are reported in Figure S3.

Moreover, flow cytometry revealed that the two highest concentrations of Cur were able to induce cell cycle arrest in the S-phase. Cur 0.05 mM and 0.1 mM induced a significant increase in the percentage of cells in the S-phase (43.3% and 53.7%, respectively) compared to the control group (17.9%). In the same experimental groups, we can also observe a subsequent significant decrease in the percentage of cells in the G0/G1 phase (35.5% and 30.7%) compared to the CTRL (61.9%) (Figure 3B,C; Table 2). Cur 0.01 mM and DMSO 1%, did not affect any phase of the cell cycle as shown in representative profiles in Figure 3A. Cell cycle analysis of ARPE-19 treated with lower concentrations of DMSO 0.1% and 0.5% did not affect cell cycle progression as well, as reported in the Supplementary Figure S2.

### 2.2. Morphological and Differentiation Analysis of Retinal Pigment Epithelial Cells after Curcumin Supplementation

**Figure 4:**
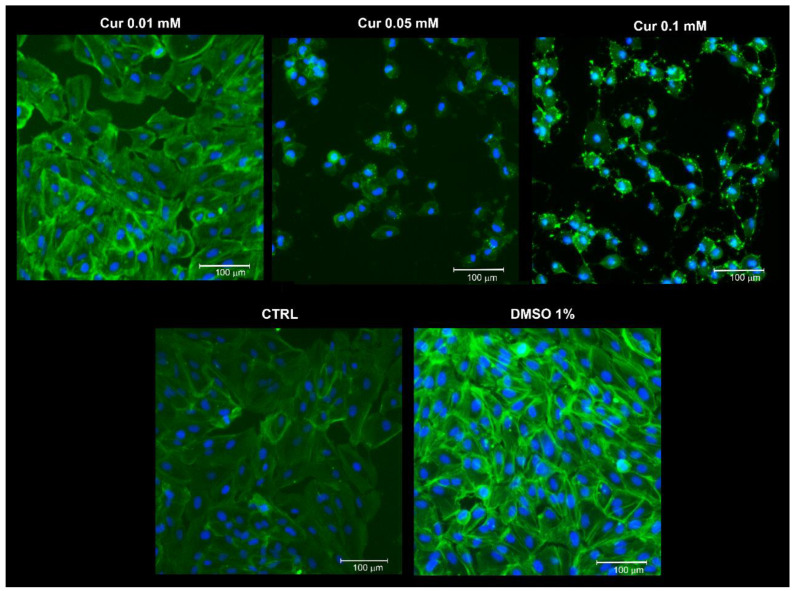
Morphological changes analysis. Representative images of ARPE-19 stained with phalloidin (green) and counterstained with the nuclear dye bisbenzimide (blue). Scale bar: 10 μm. ARPE-19 cells undergo several morphological changes, including the exhibition of cellular protrusions from the cell body that are assimilable to a neuronal-like phenotype. The images of all the vehicles groups are reported in Figure S4.

Previous studies have showed that cell cycle arrest in the S-phase could be related to neuronal or glial differentiation [27,28]. In addition, other evidence revealed the ability of ARPE-19 cells to undergo a trans-differentiation process when exposed to different substances, as reported elsewhere [29,30,31]. For this reason, we analysed cell morphology through phalloidin staining on ARPE-19 treated with an increasing concentration of Cur (Figure 4). Figure 4 shows that Cur induces cytoskeleton and morphological changes in ARPE-19 cells in a dose-dependent fashion. Particularly, samples treated with Cur 0.1 mM acquire a neuronal-like phenotype, sprouting extensions from the cell body.

**Figure 5:**
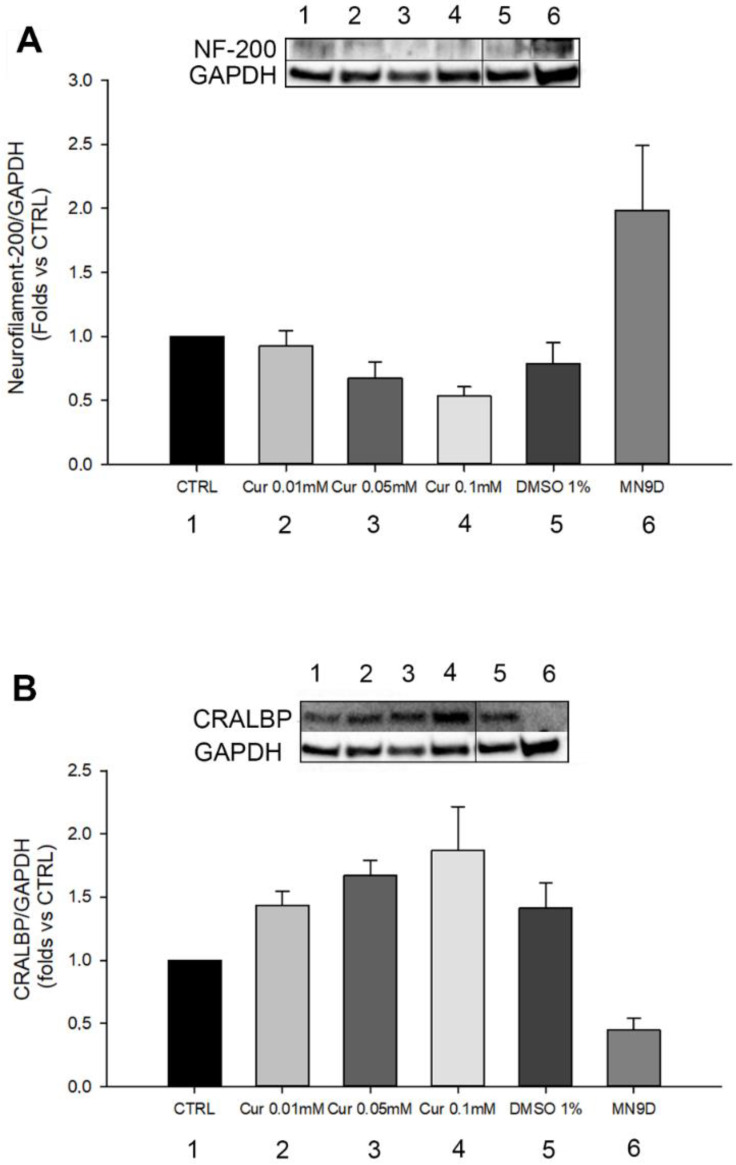
Differentiation analysis by western blot technique (**A**,**B**). Quantification of Neurofilament-200 and CRALBP protein levels in each experimental group. Statistical analysis was performed using one-way ANOVA test. No significant differences emerged from the analysis (n = 3). The expression analysis of NF-200 and CRALBP in all the vehicles groups are reported in Figure S5. Original Western Blot bands of NF-200 and CRALBP are showed in Figure S7 and Figure S8, respectively.

To confirm the hypothesis of cell differentiation, we performed a western blot of Neurofilament-200 which is important for the correct functioning of neuronal axons and was previously used to investigate ARPE-19 epithelial to neuronal differentiation [30]. We also quantified CRALBP levels, which is an RPE marker (Figure 5A,B). For both analyses, we used MN9D cells (a mouse dopaminergic cell line) as a positive and negative control, for the analysis of NF-200 and CRALBP, respectively. Previously, it has been observed that, during the differentiation process of ARPE-19 into retinal neurons, there is a decrease in the expression of CRALBP that correlates with the increase in Neurofilament-200 levels [31]. In our study, we observed that Neurofilament-200 was slightly expressed in non-treated ARPE-19 cells, as reported also in other works [30], but no changes were reported in its expression in Cur-treated cells. Moreover, we observed a slight, but not significant, increase in the protein levels of CRALBP in Cur-treated cells.

### 2.3. Electrophysiological Analysis of ARPE-19 Cells after Curcumin Supplementation

**Figure 6:**
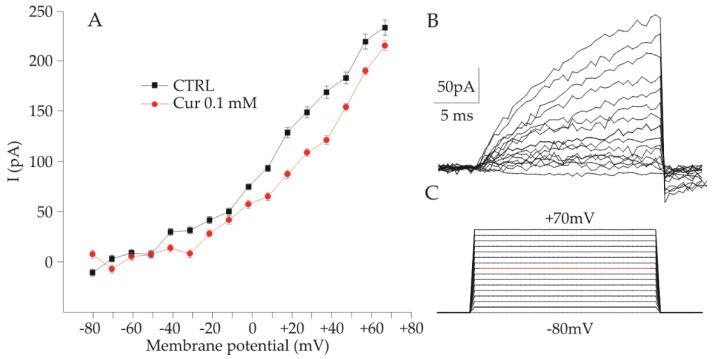
Voltage activated currents recorded in ARPE-19 cells CTRL and Cur 0.1 mM. (**A**) The currents obtained for each potential step were averaged and plotted against the respective voltage to obtain a current-voltage curve for control and curcumin treated cells. (**B**) Representative profiles of the currents obtained for each voltage steps shown in panel (**C**). (**C**) Plot of the voltage steps of 10 mV from −80 to +70 mV administered to the cell, the red line indicates 0 mV. The two curves shown in panel A are not different as assessed by the Kolmogorov-Smirnov test, suggesting that curcumin does not affect the functional properties of ARPE-19 cells.

Since it was observed that ARPE-19 cells show a neuronal-like morphology following treatment with Cur 0.1 mM, we investigated both control and treated ARPE-19 cells for the presence of voltage-gated channels, which are usually expressed in electrically excitable cells, using the patch clamp technique in the whole cell configuration. In both cases, most cells presented a resting potential, ranging from −5 to −15 mV, higher if compared with what was found in previous studies [32]. Cells were clamped at −50 mV, and all cells with a holding current of less than −500 Pa were not included in the analysis. Voltage-gated currents were elicited by voltage steps starting from −80 to +70 mV in 10 mV steps (25 ms duration), as shown in Figure 6C (the red line is relative to 0 mV). As shown in Figure 6B (upper trace), voltage steps induced outward currents both in control and treated cells. Currents started to be significantly different from the baseline for voltage steps ranging from −10 to 0 mV. No inward currents were detected. To check the nature of the outward currents, we administered both 4-aminopyridine (4-AP) 5 mM and tetraethylammonium (TEA) 10 mM, which should block voltage-gated potassium channels. Both the antagonists had little or no effect on the currents, and their nature was not further investigated. Similar results were observed under the voltage ramp protocol. The values of the currents used for the plot in Figure 6A were collected at the end of the stimulation protocol, when the response tended to a plateau, as a mean value calculated averaging the values obtained in 150 acquisition points after subtracting the baseline (i.e., the mean currents at −80 mV averaged over 400 acquisition points for each voltage step). Figure 6A shows the current/voltage relation obtained from control (n = 6 cells) and Cur treated (n = 8 cells) in separate experimental sessions. As shown in Figure 6A the two plots are very similar and by Kolmogorov-Smirnov test we obtained 0.348 as p value, indicating no statistically significant difference between the two I/V curves.

### 2.4. Curcumin Is Able to Induce Autophagic Flux Modulation in ARPE-19 Cells

**Figure 7:**
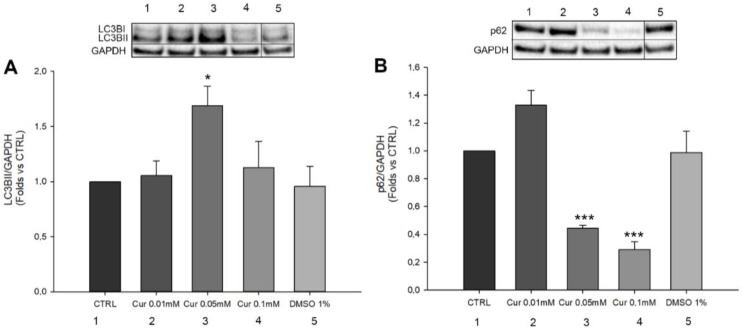
Autophagic markers quantification. (**A**,**B**) Quantification of LC3BII and p62 protein levels on ARPE-19 cells treated with Cur (0.01 mM; 0.05 mM and 0.1 mM) and DMSO 1% for 24 h. (**B**) Western Blot analysis of p62 expression on ARPE-19 cells treated with Cur and DMSO for 24 h. Statistical analysis was performed using one-way ANOVA followed by Dunnet’s test. * *p* < 0.05; *** *p* < 0.001 versus CTRL (n = 4). The expression analysis of LC3BII and p62 in all the vehicles groups are reported in Figure S6. Original Western Blot bands of LC3BII and p62 are showed in Figure S9 and Figure S10, respectively.

Autophagy is considered a dynamic process, necessary for the maintenance of cellular homeostasis; indeed, its role is the degradation of cellular components and damaged organelles in response to different environmental stimuli. Autophagy is one of the key mechanisms necessary for the proper functioning of the Retinal Pigment Epithelium (RPE). This self-eating process is involved in the degradation of photoreceptors’ outer segments and in the regeneration of visual pigments during the visual cycle [14]. In addition, recent evidence highlighted the correlation between autophagy, cell cycle, and proliferation. Particularly, autophagy induces a negative regulation of cell proliferation by inducing cell cycle arrest in response to stressful stimuli [33,34]. For this reason, we investigated whether Cur administration could modulate autophagy in ARPE-19 cells. Thus, we studied the protein levels of LC3BII and p62 through western blot analysis (Figure 7). LC3BII and p62 are widely used in autophagy. The first one is commonly used for the detection of complete autophagosome formation while the second is used to study autophagic flux exploitation. We found that Cur 0.01 mM and 0.1 mM did not induce any change in the expression of LC3BII, whereas Cur 0.05 mM induces a significant increase in the expression of LC3BII (Figure 7A). A slight but not significant increase in p62 expression was observed only after the treatment with Cur 0.01 mM, while p62 was significantly down-regulated by Cur (0.1 mM and 0.05 mM) (*p* < 0.001 in both cases) compared to the control (Figure 7B). DMSO 1% did not induce any changes in the expression of both LC3BII and p62. 

## 3. Discussion

Curcumin is a natural and biologically-active molecule that has been largely studied because of its versatility; indeed, there is a huge number of studies based on the use of curcumin in different pathological contexts, including ocular diseases [35,36]. This compound has been shown to elicit antioxidant, anti-inflammatory, and anti-angiogenic effects in various cell systems [36]. On the other hand, some investigations reported possible cytotoxic effects of curcumin, including the blocking of cell proliferation, cell cycle arrest, apoptotic cell death, and pro-oxidative effects. These studies also underline that the protective or cytotoxic outcomes of curcumin are strongly related to the dose used [7,8,37]. For this reason, shedding light on the mechanisms by which this compound acts is crucial from a translational point of view. In this work, we focused our interest on the specific mechanisms triggered by curcumin supplementation on Retinal Pigment Epithelial cells (ARPE-19), a widely used cell line for in vitro studies of treatments/drugs [19,21,38,39,40,41]. We compared the effects of three different concentrations of curcumin.

The first step of our study was to investigate whether curcumin treatment could affect cell proliferation. We showed a dose-dependent effect of curcumin on ARPE-19 proliferation; indeed, the two highest doses used (0.05 mM and 0.1 mM) induced a significant reduction in cell viability compared to the CTRL t24, while the lower dose induced a significant increase in cell proliferation compared to the CTRL. In order to understand if the observed effects were assimilable to cell death or proliferation arrest, we added another control in the experiment, called CTRL t0, through which we underlined the antiproliferative effects of curcumin on ARPE-19 cells. The ability of curcumin to affect cell proliferation has already been assessed in different cell types and our data are consistent with those reported elsewhere [7,24,36]. On the other hand, we observed an increase in cell proliferation after the treatment with Cur 0.01 mM. This evidence is in agreement with data reported elsewhere [35] and confirmed the opposing dose-dependent effects of curcumin on RPE cells. By focusing on the ability to promote cell viability, it is known that Curis was able to modulate the ratio between Bax and Bcl-2, preventing apoptotic cell death in different models, including ARPE-19 cells [42,43]. Based on our evidence of the impact of curcumin on cell proliferation, we performed flow cytometry analysis to evaluate apoptosis levels and cell cycle progression. No changes emerged from the study of apoptosis levels. By contrast, we pointed out a marked effect of curcumin on the progression of the cell cycle, highlighting the S-phase arrest in ARPE-19 cells treated with Cur 0.5 mM and 0.1 mM. The cell cycle is a finely regulated process involved in proliferation, stemness, differentiation, and development [44]. Our data are in line with the literature since previous studies demonstrated multiple alterations in cell cycle progression in various types of cancer cell lines, in human umbilical vein endothelial cells, and also in retinal pigment epithelial cells upon Cur treatment [25,45]. Furthermore, similar features have been associated with neuronal and glial differentiation in different cellular models as reported elsewhere [27,28]. Previous studies have already shown that ARPE-19 cells could trans-differentiate into retinal neuronal cells under certain stimuli because of the common embryonal origins of RPE and neural retina. Particularly, the supplementation of Fenretinide (a derivate of the retinoic acid), or bFGF, induced the acquisition of a neuronal-like phenotype [29,30]. We observed that Cur-treated ARPE-19 cells show morphological changes assimilable to the trans-differentiation process reported elsewhere [30]. For this reason, we explored a possible differentiation process through morphological, molecular, and functional analysis but we did not observe significant differences between treated and untreated cells. We used the patch clamp technique in whole-cell configuration to investigate the presence of voltage-gated channels in ARPE-19 cells, both native and upon 24 h treatment with Cur 0.1 mM, to assess if their morphology was associated with the neuronal-like functional phenotype. We tested cells with step potentials (−80 to +70 mV) and ramps (−100 to 100 mV). Both protocols only elicited outward currents that were only slightly affected by TEA or 4-AP, which were used to evaluate the possibility that the current could be due to potassium voltage-gated channels. Since 4-AP and TEA insensitive channels have already been described in pigmented ciliary epithelial cells [32], the activation of voltage-dependent potassium channels cannot be excluded. Recently, the presence in ARPE-19 cells of both cationic and anionic depolarizing voltage-induced currents was described, and current values at +70 mV were found compatible with those observed in our experiments [46]. Therefore, we can speculate that our results may derive from both chloride and potassium currents, thus further experiments are necessary to assess this issue. In conclusion, our results suggest that the morphological features observed under Cur 0.1 mM are not compatible with a functional neuronal phenotype, in agreement with what was observed from the protein quantification of Neurofilament-200. As a consequence, we can affirm that ARPE-19 cells treated with curcumin were not differentiating.

However, in order to understand the mechanism involved in proliferation and cell cycle arrest, we investigated the autophagy process. Recently, some studies underlined the emerging role of autophagy in the regulation of cell cycle progression and arrest [33,34]. Furthermore, autophagy plays a pivotal role in the correct functioning of the visual system, allowing the digestion and recycling of components resulting from the shedding of the photoreceptors outer segments (POS). RPE is constantly exposed to environmental stressors and the proper regulation of the autophagic machinery is fundamental for their function; in fact, autophagy impairment due to aging or pathological events leads to the degeneration of RPE cells [14]. We analysed LC3B and SQSTM1/p62, and we observed a significant increase in the expression of LC3BII only in samples treated with curcumin 0.05 mM, while the lower and the higher concentrations used did not affect its expression. On the other hand, we found a significant reduction of SQSTM1/p62 in cells treated with 0.05 mM and 0.1 mM of curcumin, and no significant differences in those treated with the lower concentration. Although the protein levels of LC3BII per se did not give us clear information about autophagy activation/inhibition, its correlation with SQSTM1/p62 expression suggests an important activation of this process. This interpretation is in agreement with the guidelines for the use and interpretation of assays for monitoring autophagy which indicates that the amount of LC3BII may be not directly related to the activation of autophagy and its expression could change in an unpredictable way [47]. Furthermore, the fact that we observed the modulation of autophagy, and the arrest of the cell cycle leads us to assume that the correlation between these two processes may be the key to interpreting our data. 

The complex interaction between autophagy and the cell cycle has been recently addressed. Previous studies have shown that this ‘self-eating’ process is differentially regulated in each phase of the cell cycle and its activation occurs mainly in the G1 and S phases [33]. These two important cellular processes play a homeostatic role in the physiological condition, but they can modulate their activity in relation to stressful stimuli, in which their correlation is more evident. Indeed, factors involved in cell cycle arrest as p21, p16, and p27 also activate autophagy under certain circumstances. Autophagy could negatively regulate proliferation and induce cell cycle arrest as a cytoprotective effect after DNA damage [48]. It is also worth noting that curcumin is able to induce DNA damage; particularly, several pieces of evidence exist about its capability to induce Double Strand Brakes (DSB) in different cancer cell lines [37]. Even though less is known about RPE cells, some studies demonstrated the ability of curcumin to induce DNA fragmentation in hRPE cells [7,8]. Furthermore, through confocal microscopy, we observed that in ARPE-19 cells, Cur tends to accumulate in the nuclei of the cells (Supplementary Figure S11). Thus, it may be reasonable to believe that in ARPE-19 cells, the highest concentrations of curcumin used in this work could cause DNA damage. This is coherent with the indication of S-phase arrest and autophagy activation since the former gives more time to cells to implement repair mechanisms of DNA and the latter is known to be important in DNA Damage Response (DDR) since its inhibition leads to apoptotic cell death [48]. In this context, SQSTM1/p62 could be considered the key mediator between autophagy and DNA repair mechanisms. Indeed, it was observed that a decrease in SQSTM1/p62 protein levels is related to the activation of DDR and DNA repair systems [49]. Within this framework, we can suppose that S-phase arrest and autophagy activation could represent two mechanisms through which RPE cells reply to stressful stimuli induced by high doses of curcumin. This interpretation is in accordance with previous evidence about the cytotoxic effects of curcumin in particular conditions and contributes to underlining new insights into the effects of this very useful compound. 

## 4. Materials and Methods

### 4.1. Cell Culture

ARPE-19 (Adult Retinal Pigment Epithelial cells) cell line was purchased by the American Type Culture Collection (ATCC, Manassas, VA, USA). Cells were cultured in a medium containing a mixture of Dulbecco’s Modified Eagle Medium (DMEM) and Ham-F12 (1:1) (Gibco, Thermo-Fisher Scientific, Monza, Italy) and supplemented with 10% Fetal Bovine Serum (FBS) (Corning, New York, NY, USA), 1% Glutamine, and 1% Penicillin/Streptomycin (Gibco, Thermo-Fisher Scientific, Monza, Italy). Cells were grown in a humidified atmosphere at 37 °C with 5% CO_2_. Cells were used between passages 6 and 19.

### 4.2. Curcumin Treatment

Curcumin (Linnea, cat# CUM 1259) was dissolved in DMSO (MP Biomedicals, USA) to obtain a starting concentration of 10 mM. The treatment of ARPE-19 cells was then performed with three different concentrations of curcumin: 0.01 mM, 0.05 mM, and 0.1 mM for 24 h. DMSO 0.1%, 0.5%, and 1% were used as the vehicle. 

### 4.3. Cell Viability Assay

**Figure 8:**
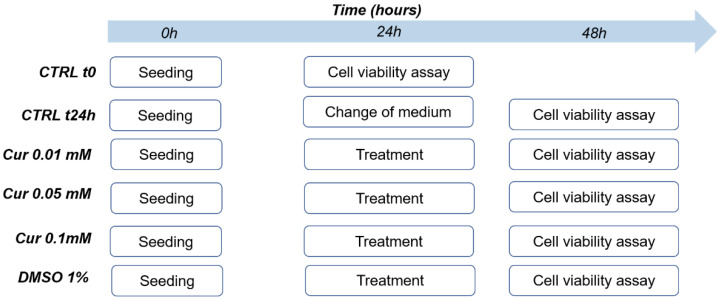
Scheme of the experimental flow used during the cell viability assay.

Cell Viability was evaluated by Cell Counting Kit-8 (CCK8, Sigma Aldrich, Saint Louis, MO, USA) according to the manufacturer’s protocols. ARPE-19 cells were seeded at a density of 15 × 10^3^/well in 96-well microplates (Corning, USA) for 24 h before the curcumin and vehicle treatments. After 24 h, 10 μL of CCK8 reagent was added to each well and incubated in a humidified atmosphere at 37 °C and 5% CO_2_ for 4 h. The absorbance was read at 450 nm with a microplate reader (Infinite M Plex, Tecan, Switzerland) as showed in Figure 8. 

The experimental groups included in this study are reported below: 

Ctrl t0: ARPE-19 cells analysed 24 h after the seeding, that is before the start of Cur treatment. 

Ctrl 24 h: ARPE-19 cells treated with complete medium and analysed at the end of the experiment.

Cur 0.01 mM: ARPE-19 cells treated with curcumin [0.01 mM] and analysed after 24 h.

Cur 0.05 mM: ARPE-19 cells treated with curcumin [0.05 mM] and analysed after 24 h.

Cur 0.1 mM: ARPE-19 cells treated with curcumin [0.1 mM] and analysed after 24 h.

DMSO 1%: ARPE-19 cells treated with DMSO 1% and analysed after 24 h. 

### 4.4. Phalloidin Staining

To evaluate any morphological changes induced by the treatments, phalloidin staining was performed for each experimental group. For this purpose, ARPE-19 cells were seeded at 2 × 10^5^ cells/well density in 6-well plates. Cells were washed two times with Phosphate-Buffered Saline (PBS), were fixed with 4% Paraformaldehyde (PFA) for 10 min, and were incubated with 3% Bovine Serum Albumin (BSA) with 0.1% Triton for 30 min for non-specific binding sites block. Fluorescein Isothiocyanate conjugated Phalloidin (Sigma Aldrich, Saint Louis, MO, USA) (1:250 in PBS1X), incubated for 40 min at Room Temperature (RT), was used to label cellular cytoskeleton and Bisbenzimide to counterstain cellular nuclei. Images were acquired with Floid Cell Imaging Station (Thermo Fisher Scientific Inc., Monza, Italy). 

### 4.5. Flow Cytometer Analysis

Flow cytometry and Propidium Iodide (PI) staining were used to carry out cell cycle and apoptosis analysis. Cells at 1 × 10^6^ density were seeded in 10 cm dishes for 24 h and then treated with Curcumin (0.01 mM, 0.05 mM, and 0.1 mM) and DMSO (0.1%, 0.5%, and 1%) as the vehicle for 24 h. Cells were incubated with 1X trypsin in a humidified atmosphere at 37 °C with 5% CO_2_ for 5 min and then centrifuged (1300 rpm for 10 min at 4 °C). Cells were fixed with ice-cold ethanol (70% in PBS) for 30 min at 4 °C and then were transferred to plastic BD tubes (Becton Dickinson, San José, CA, USA), washed twice with the ice-cold PBS, and stained with a mixture solution of PI (50 µg/mL), Nonidet-P40 (0.1% *v*/*v*), and RNase A (6 µg/10^6^ cells) (all from Sigma-Aldrich, Saint Louis, MO, USA) for 1 h in the dark at 4 °C. The percentages of cells in the G1, G2/M, and S phases (data from 10,000 events per sample) were calculated by the software Modfit LT for Mac V3.0 using a FACS Calibur instrument (BD Biosciences). The assay was carried out in duplicate. Data from 10,000 events per sample were collected and analyzed. The apoptotic cells were determined by their hypochromic subdiploid nuclei staining profiles and analyzed while using the Cell Quest software program (BD Instruments Inc., San José, CA, USA).

### 4.6. Western Blot 

1.1 × 10^6^ cells were cultured for 24 h in a 10 cm petri dish and then treated as described above. Afterwards, cells were lysed using a lysis buffer containing 50 mM Tris-HCl pH 7.5, 1% Triton x-100, 0.1% SDS, ethylenediaminetetraacetic acid EDTA 5 mM, Halt Protease and Phosphatase Inhibitor cocktail (Thermo Fisher Scientific Inc., Monza, Italy). Bradford assay was used to evaluate the protein quantity and then 35 μg of total proteins were run on a Bolt 4–12% Bis-Tris Plus (Thermo Fisher Scientific Inc., Monza, Italy) at 200 V for 20 min. Additionally, proteins were transferred on a PVDF membrane by the iBlot 2 Dry Blotting System (Invitrogen, Waltham, MA, USA). 5% non-fat dry milk in TBST (Tris Buffered Saline with 0.1% Tween) was used to block non-specific binding sites at RT for 1 h; then the membranes were incubated with different primary antibodies in 5% non-fat dry milk in TBST overnight at 4 °C: anti-LC3BII (1:1500) (Thermo Fisher scientific Inc, Monza, Italy) and anti-p62 (1:1000) (Cell Signaling) for the study of autophagy pathway; anti-GAPDH (1:1500) (Thermo Fisher Scientific Inc, Monza, Italy); anti-CRALBP (1:1000) (Thermo Fisher scientific Inc., Monza, Italy) as an RPE marker and anti-Neurofilament-200 (1:1000) (Abcam, Prodotti Gianni, Milan, Italy) as a marker of neuronal differentiation as described elsewhere [30]. Next, the membranes were washed two times and incubated with anti-rabbit or anti-mouse HRP (Horseradish Peroxidase)-conjugated secondary antibodies in 5% non-fat dry milk in TBST for 45 min at RT. The signal was detected using SuperSignal West Pico Plus (Thermo Fisher Scientific Inc., Monza, Italy) substrates and ChemiDoc XRS plus imaging system (Bio-Rad Laboratories, Milan, Italy). The densitometric analysis was performed by ImageJ software. 

### 4.7. Patch Clamp Recordings

1 × 10^5^ ARPE-19 cells were seeded in a 35 mm petri dish and grown for 24 h. Then we treated the cells with fresh medium for the CTRL group and with Cur 0.1 mM for 24 h before recordings. Before the measurements, the culture medium was removed and replaced with the recording solution. Cells were positioned on the stage of an upright microscope Axioskop FS2 (Zeiss, Gottingen, Germany), visualized by a 40× objective and continuously superfused by a gravity-driven perfusion system connected to a four ways perfusion pipette positioned at the border of the visualized microscope field. The recording and reservoir solution was composed of 140 mM NaCl, 2.5 mM KCl, 2 mM MgCl_2_, 2 mM CaCl2, 10 mM HEPES, and 10 mM glucose, adjusted to pH 7.3 with NaOH. Drugs, 4-aminopyridine (4-AP) 5 mM and tetraethylammonium (TEA) 10 mM were added to perfusion reservoirs and administered by switching from the control solution. Patch clamp electrodes with a resistance of 3 to 5 MW were pulled from borosilicate glass using a PC-10 puller (Narishige, Japan). The electrodes were filled with a solution containing 140 mM KCl, 2 mM MgCl2, 0.5 mM EGTA, 2 mM NaATP, and 10 mM HEPES, adjusted to pH 7.3 with KOH. Whole-cell currents were measured using an EPC-7 patch-clamp amplifier (Heka, Lambrecht, Germany) connected to a NI BNC-2090 acquisition board (National Instrument, CA, USA), low pass filtered (3–5 Khz), acquired with WinWCP (J. Dempster, University of Strathclyde, Glasgow, UK) and analyzed with Clampfit software (Axon Instruments, Foster City, CA, USA). Fast and slow capacity transients were compensated. All experiments were performed at room temperature (22–25 °C). Leak-subtracted data within WinWCP were plotted using SIGMAPLOT software (Palo Alto, CA 94303, USA). All reagents for electrophysiology were from SIGMA (Merk Life Science S.r.l., Milano, Italia).

### 4.8. Statistical Analysis

All data are presented as mean ± SE of a minimum of three independent experiments. Statistical analysis was performed using a one-way ANOVA test. Post hoc comparisons were made through Dunnet’s test. To evaluate the difference in the proliferation rate between the treated groups and CTRL t0, we provided estimates of the mean differences with their standard errors. The most relevant estimates were reported in the result section. The Kolmogorov-Smirnov test was used for the statistical analysis of patch clamp data. The first type error was set at 5%. The Statistical analysis was performed using SigmaPlot 14.0 software. 

## 5. Conclusions

Taken together our results agree with the high versatility of Curcumin for application in ophthalmic diseases. Indeed, in ocular pathologies with uncontrolled and pathological RPE proliferation, such as adenocarcinoma of RPE [50] and proliferative vitreoretinopathy [3], administration of high concentrations of Cur may be recommended to limit the progression of the disease. By contrast, a lower dosage of Cur should be used for ocular pathologies characterized by RPE degeneration, such as Age-related macular degeneration (AMD) [1], to promote RPE health and survival. We also showed new dose-dependent effects of curcumin, highlighting mechanisms by which curcumin acts on RPE cells which lays the foundation for further studies in the field. 

## Figures and Tables

**Table 1:** In the table is reported the mean ± SE of the apoptosis rate in ARPE-19 cells treated with Cur (0.01 mM, 0.05 mM and 0.1 mM) and DMSO 1%. Values represents the percentage of apoptosis for each experimental group. The apoptosis rate of all the vehicles groups is reported in the Supplementary Table S1.

Groups	Apoptosis Rate
CTRL	0.6 ± 0.35
Cur 0.01 mM	0.3 ± 0.3
Cur 0.05 mM	0.4 ± 0.06
Cur 0.1 mM	0.4 ± 0.12
DMSO 1%	0.3 ± 0.03

**Table 2:** In the table is reported the mean ± SE. Values represents the percentage of cells in each phase of the cell cycle. * Significance of statistical analysis performed through one-way ANOVA followed by Tukey test. *p* < 0.001 in all the groups marked with * versus CTRL. The percentage of cells distributed in cell cycle phases of all the vehicles are reported in Supplementary Table S2.

Groups	G0/G1	S	G2/M
CTRL	61.98 ± 2.89	17.92 ± 3.19	20.09 ± 0.30
Cur 0.01 mM	65.1 ± 1.02	17.82 ± 3.84	16.87 ± 2.81
Cur 0.05 mM	33.48 ± 2.83 *	43.17 ± 0.75 *	23.36 ± 3.58
Cur 0.01 mM	30.72 ± 1.45 *	53.75 ± 3.23 *	15.53 ± 4.64
DMSO 1%	72.23 ± 1.47	11.94 ± 2.45	15.84 ± 0.99

## Data Availability

Data is contained within the article or in the Supplementary Material.

## Supplementary Materials

The following supporting information can be downloaded at: https://www.mdpi.com/article/10.3390/ijms232314771/s1.
