# Supplementary material for: New Insights into Dose-Dependent Effects of Curcumin on ARPE-19 Cells
Source: Int J Mol Sci. 2022 Nov 25;23(23):14771. doi: 10.3390/ijms232314771 (PMC9738655; doi:10.3390/ijms232314771)
Supplement: Supplementary file 1 [file ijms-23-14771-s001.zip › ijms-2029297-supplementary.pdf]

Supplementary Figures

Supplementary Figure 1

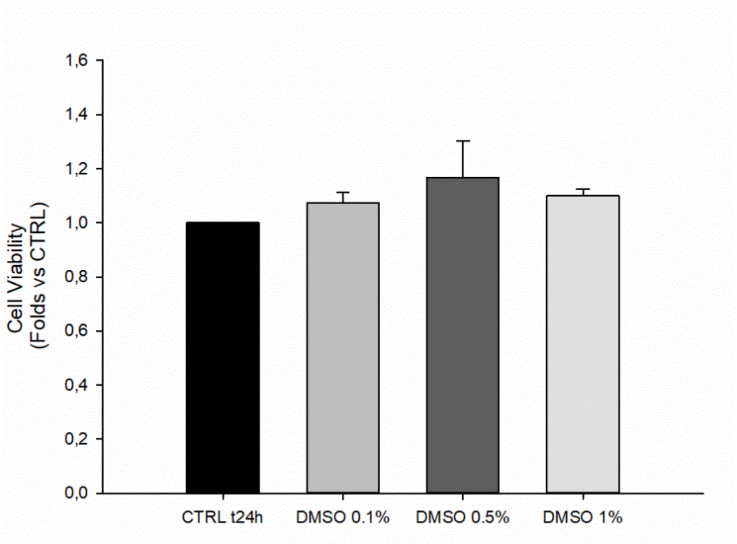

Figure S1. Effects of DMSO (used as a vehicle) on ARPE-19 cells. Cell viability of ARPE-19 cells treated with three different percentages of DMSO (0.1%, 0.5% and 1%). No significant differences are obtained between DMSO-treated and untreated cells (CTRL t24h). Statistical analysis was performed using one way ANOVA test.

Supplementary Figure 2

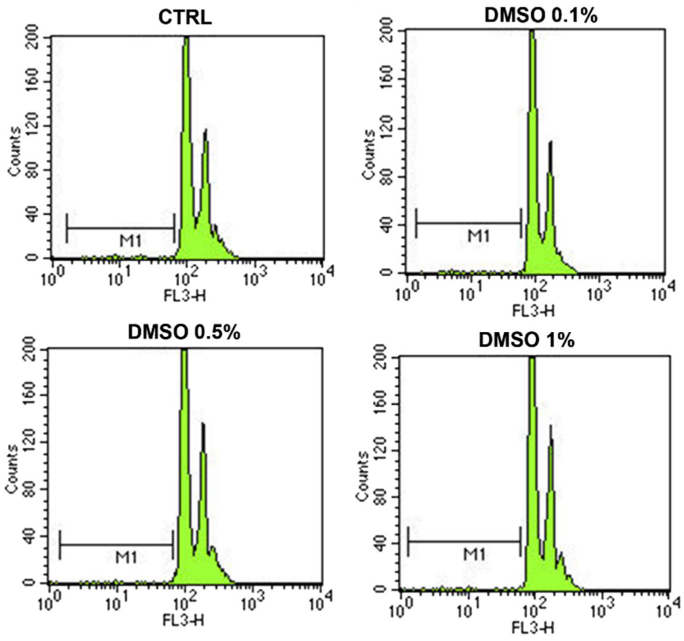

Figure S2. Flow cytometry analysis of apoptosis rate in ARPE-19 cells in presence or absence of DMSO (0.1%, 0.5% and 1%).

Groups	Apoptosis rate
CTRL	0.6±0.35
DMSO 0.1%	0.4±0.07
DMSO 0.5%	0.3±0.01
DMSO 1%	0.4±0.03

Supplementary Table S1. In the table are reported the means±SE of experimental groups treated with DMSO 0.1%, 0.5% and 1%. Statistical analysis was performed using one way ANOVA. No significant differences emerged between groups.

Supplementary Figure 3

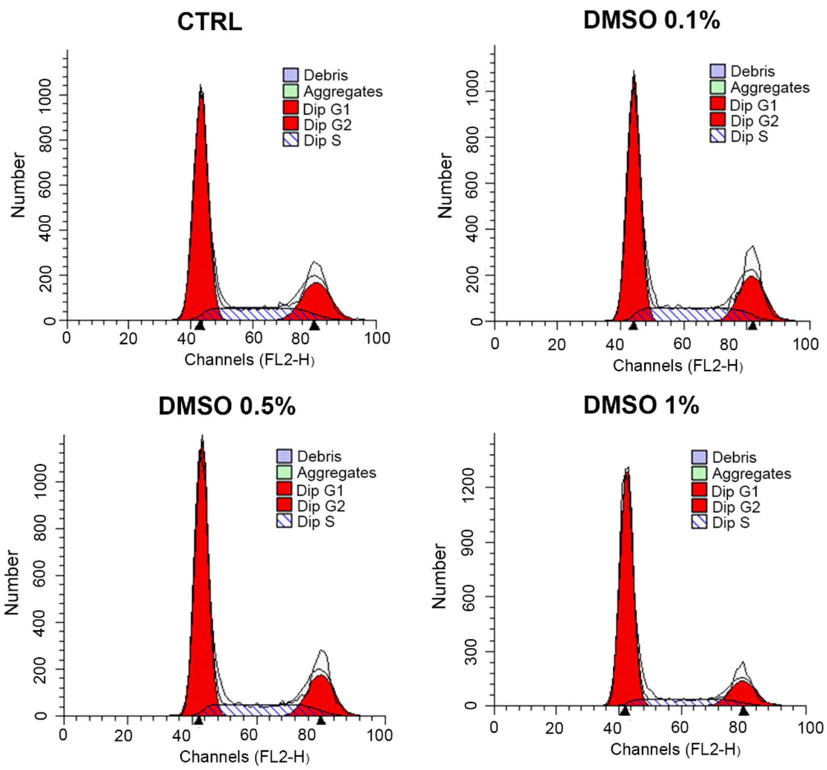

Figure S3. Flow cytometry analysis of cell cycle. Representative profiles of the distribution in the cell cycle of ARPE-19 cells in all experimental groups: CTRL, DMSO 0.1%, 0.5% and 1%. Debris and aggregates were not detected.

Groups	G0/G1	S	G2/M
CTRL	61.9±2.89	17.9±3.19	20.1±0.30
DMSO 0.1%	59.5±2.60	18.3±3.95	22.2±1.28
DMSO 0.5%	65.3±3.03	15.5±3.50	19.2±0.50
DMSO 1%	72.2±1.47	11.9±2.45	15.8±0.99

Supplementary Table S2. In the table are reported the percentages of cells in each phase of the cell cycle of all experimental groups The values represent. Statistical analysis was performed using one way ANOVA test followed by Holm-Sidak test. No significant difference emerged from the analysis.

Supplementary Figure 4

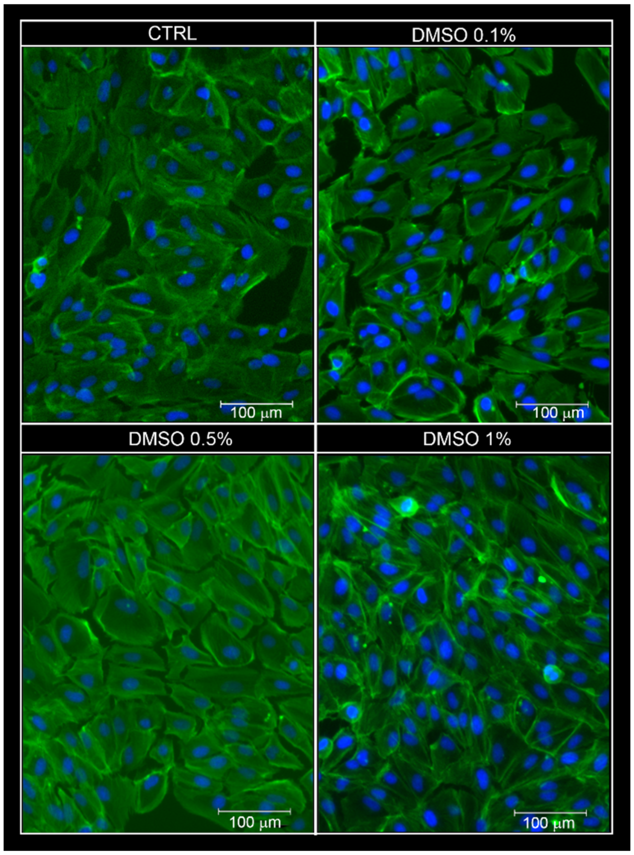

Figure S4. Phalloidin staining of ARPE-19 treated with DMSO 0.1%, 0.5% and 1%. No morphological changes were observed in the treated cells compared to the control group (CTRL).

Supplementary Figure 5

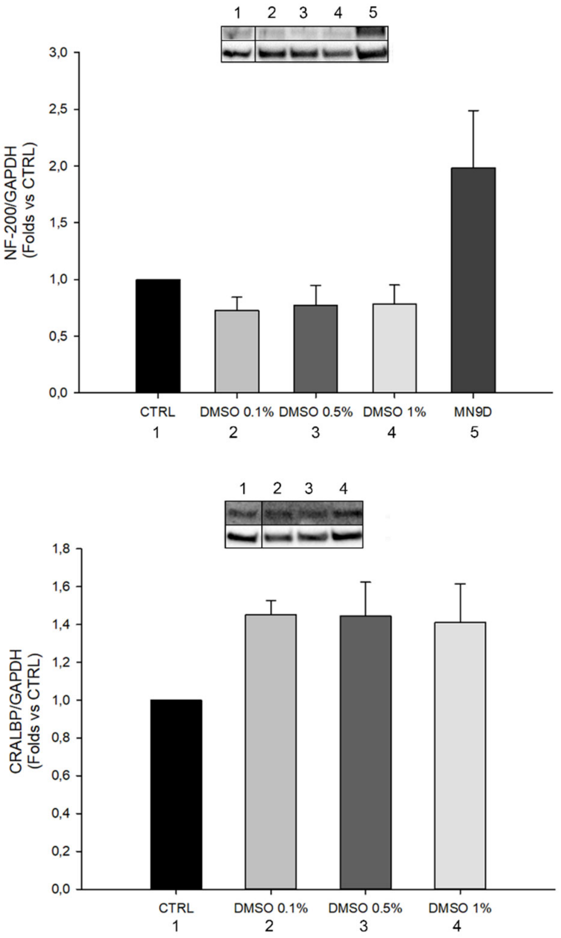

Figure S5. Western Blot analysis of NF-200 (neuronal marker) and CRALBP (epithelial cells marker) in ARPE-19 cells no treated (CTRL), and treated with DMSO (0.1%, 0.5%, 1%). Statistical analysis was performed using one way ANOVA test and no significant differences were detected.

Supplementary Figure 6

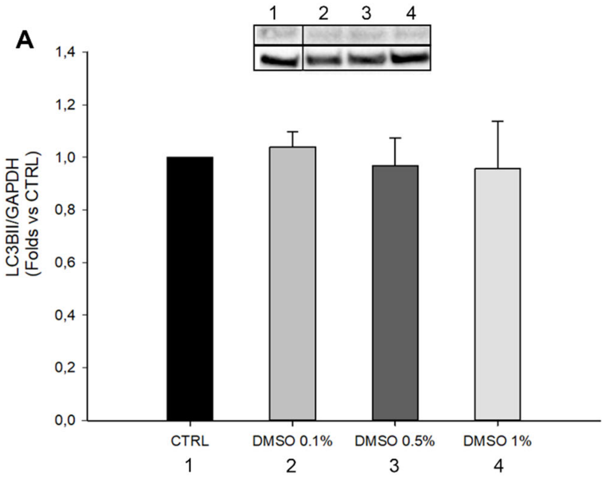

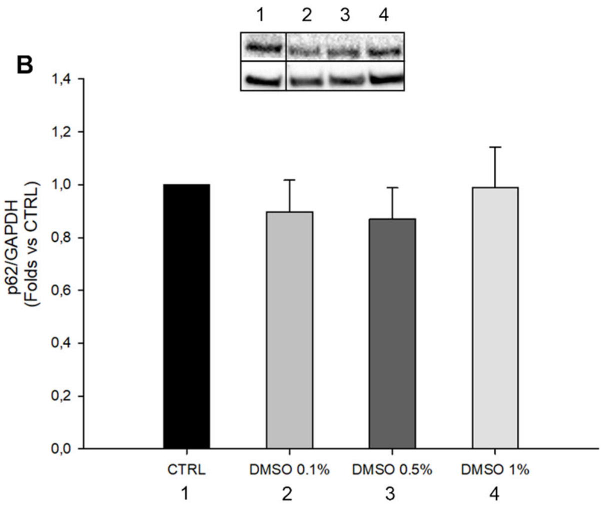

Figure S6. Western blot analysis of LC3BII and p62 in no treated ARPE-19 cells (CTRL), and treated with DMSO (0.1%, 0.5%. 1%). Statistical analysis was performed using one way ANOVA test but no significant differences were detected in the expression of LC3BII and p62.

Supplementary Figure 7

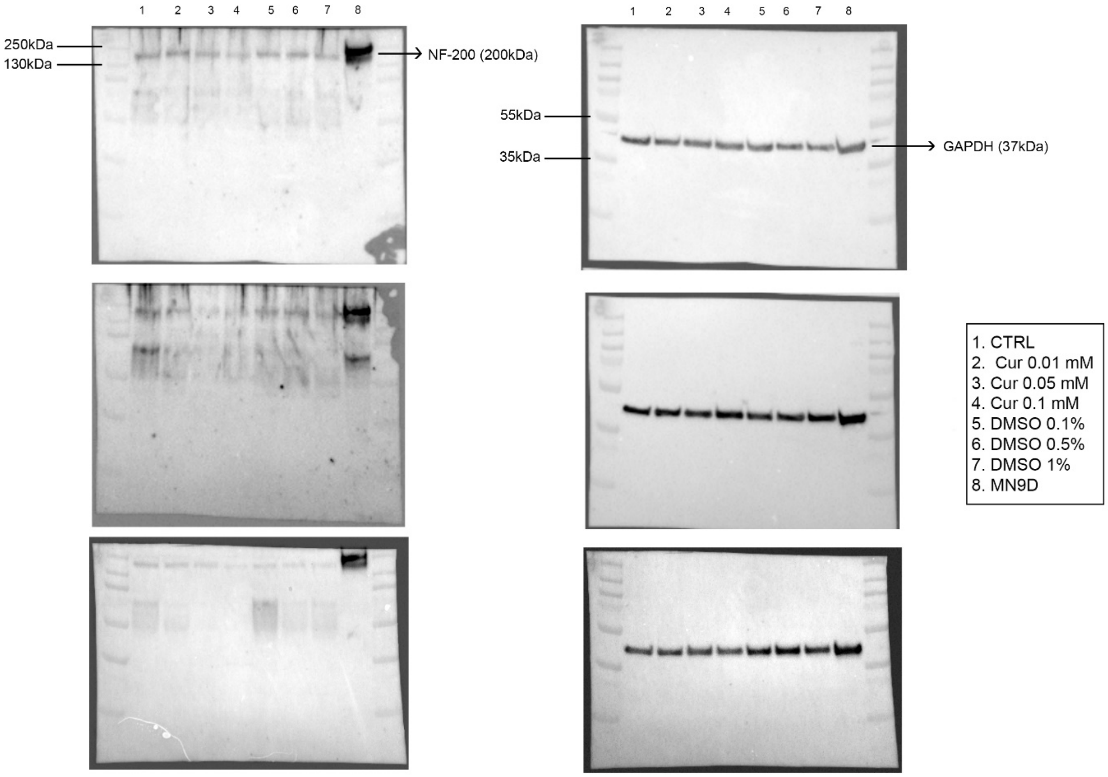

Figure S7. Whole western blot images used for the identification and quantification of NF-200 and GAPDH. The blot images were merged with the images of molecular weights in order to identify the bands for the subsequent analysis. The legend shows the experimental groups numbering; each number represents their collocation on the gel.

Supplementary Figure 8

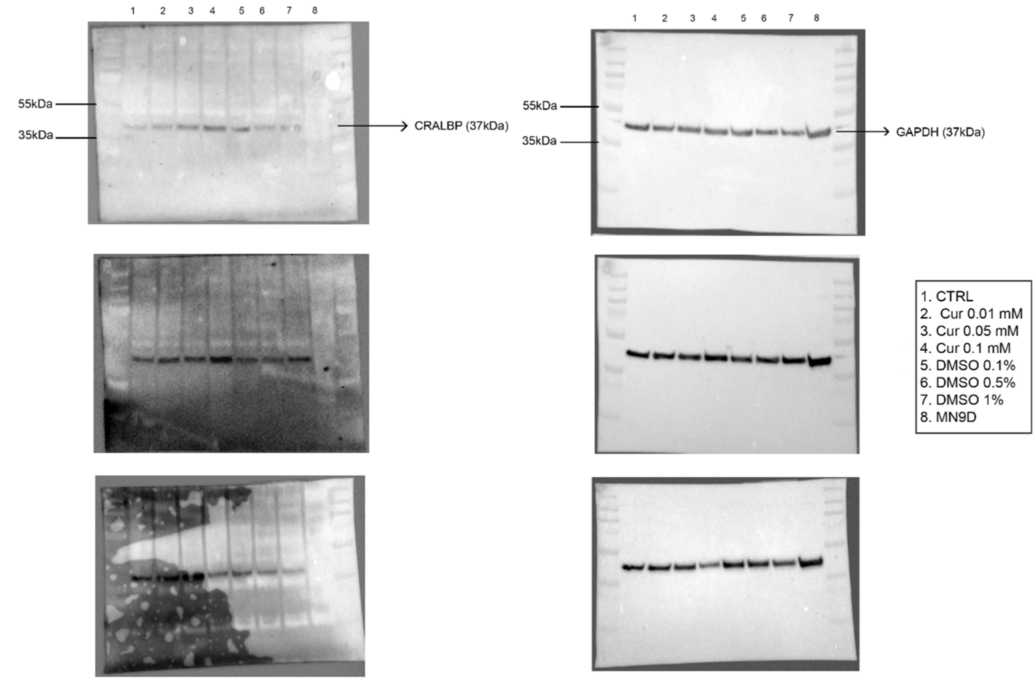

Figure S8. Whole western blot images used for the identification and quantification of CRALBP and GAPDH. The blot images were merged with the images of molecular weights in order to identify the bands for the subsequent analysis The legend shows the experimental groups numbering; each number represents their collocation on the gel.

Supplementary Figure 9

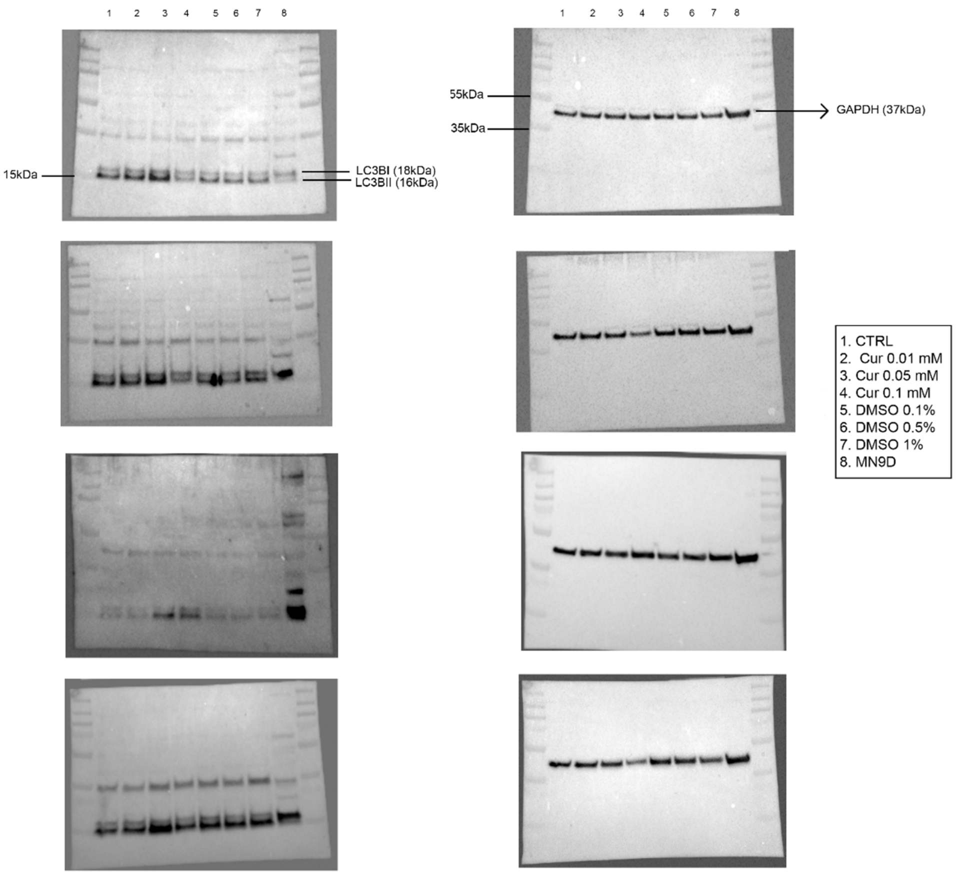

Figure S9. Whole western blot images used for the identification and quantification of LC3BII and GAPDH. The blot images were merged with the images of molecular weights in order to identify the bands for the subsequent analysis. The legend shows the experimental groups numbering; each number represent their collocation on the blot.

Supplementary Figure 10

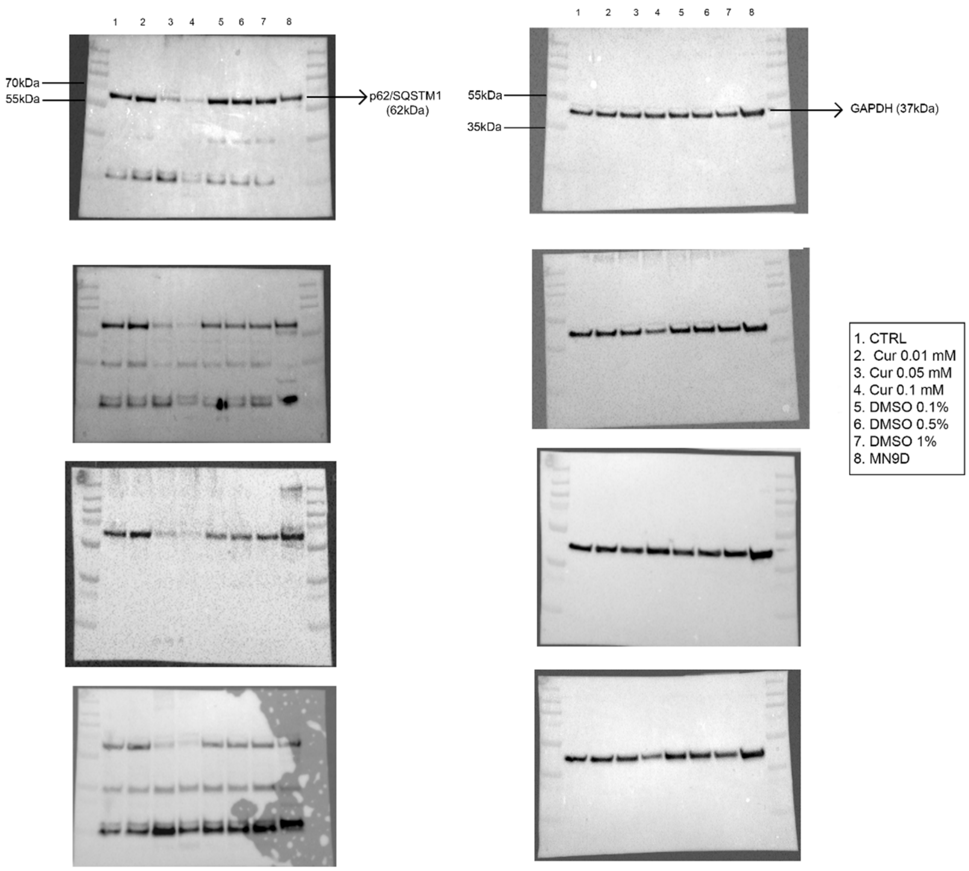

Figure S10. Whole western blot images used for the identification and quantification of p62/SQSTM1 and GAPDH. The blot images were merged with the images of molecular weights in order to identify the bands for the subsequent analysis. The legend shows the experimental groups numbering; each number represent their collocation on the gel.

Supplementary Figure 11

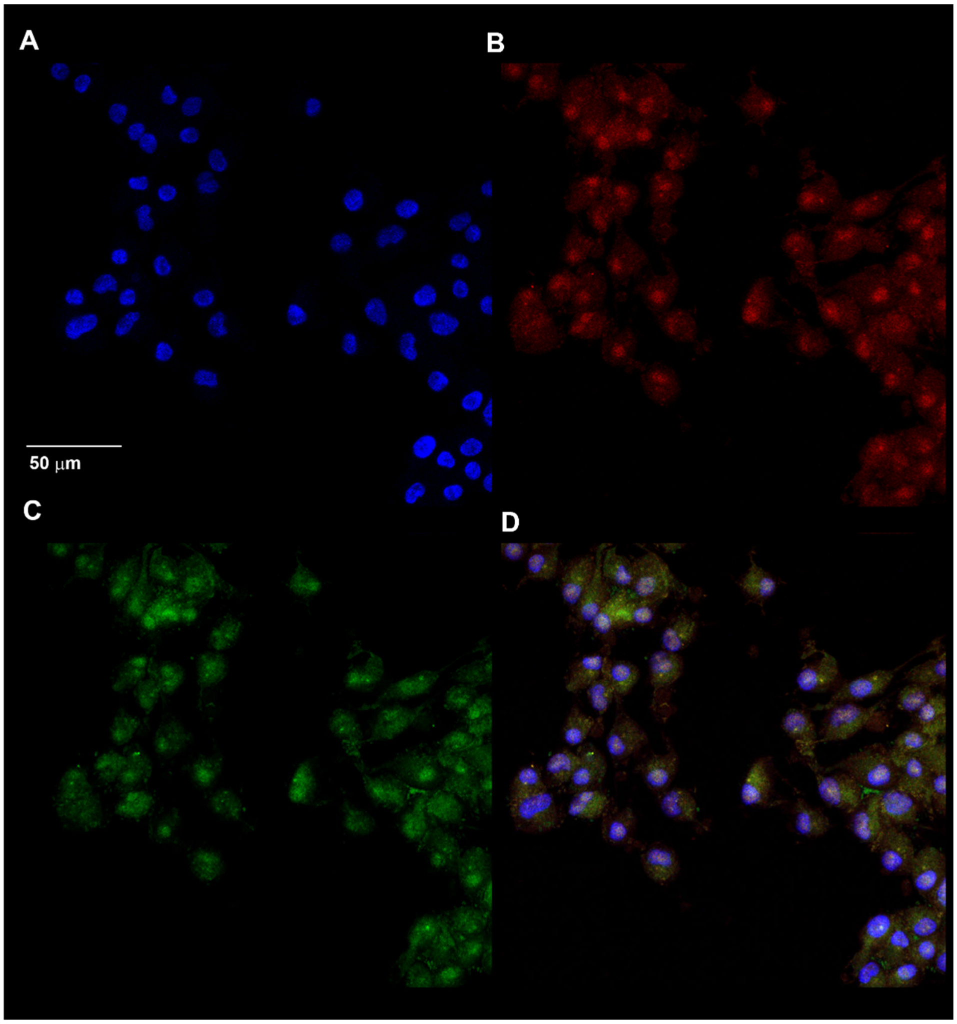

Figure S11a. Curcumin was found in the nuclei and cytoplasm of ARPE-19 treated cells because of its autofluorescence. In this image are reported confocal acquisitions of ARPE-19 cells treated with curcumin 0.1 mM and stained with bisbenzimide (a nuclear dye). (A) Blue: nuclei stained with bisbenzimide in ARPE-19 cells treated with curcumin 0.1 mM; (B, C) curcumin localization in ARPE-19 cells. (B) Red: emission of curcumin autofluorescence in the red spectrum; (C) green: emission of curcumin autofluorescence in the green spectrum. (D) merge of the three images through which we observed the colocalization of curcumin autofluorescence and bisbenzimide in the nuclei of the treated cells.

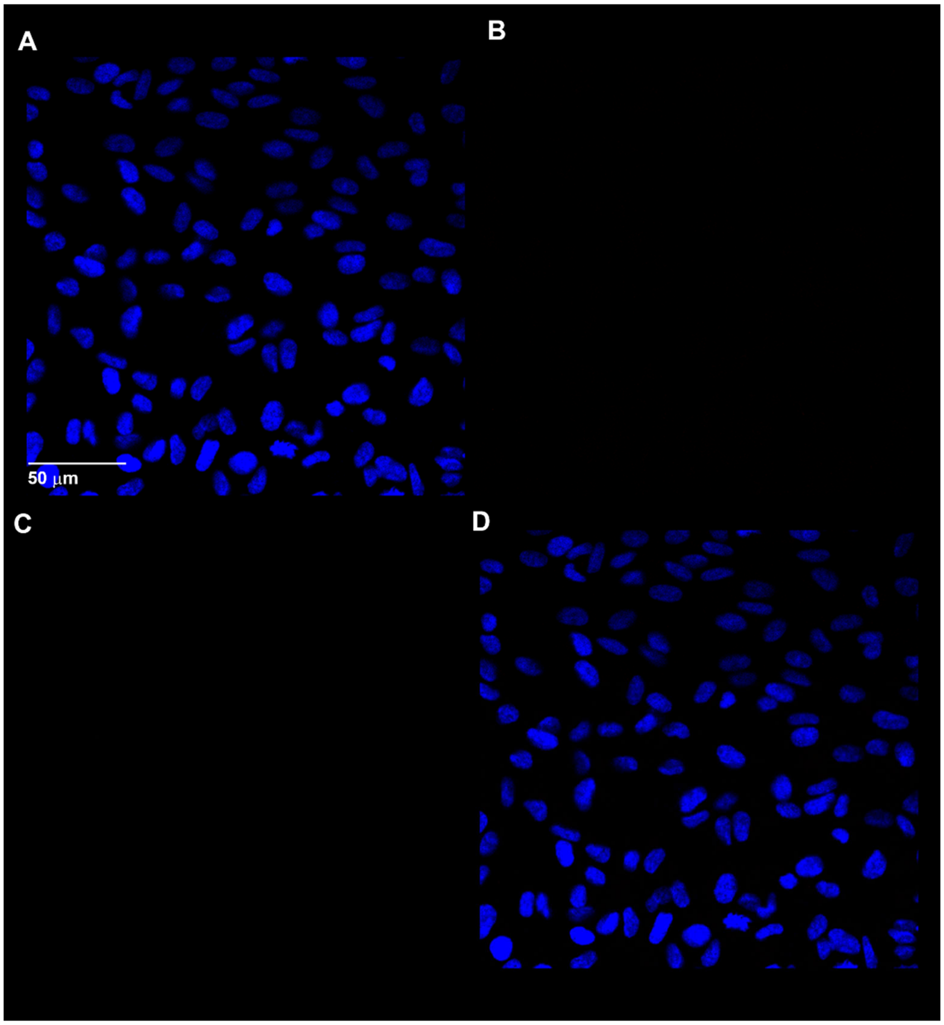

Figure S11b. ARPE-19 cells stained with bisbenzimide (a nuclear dye) used as negative control. For the acquisition of these images, we used the same parameters used in Figure S11a but no autofluorescence was detected in CTRL cells. (A) Blue: nuclei stained with bisbenzimide in CTRL ARPE-19 cells; (B) emission of non-treated cells in the red spectrum (594 nm excitation wavelength); (C) emission of non-treated cells in the green spectrum (488 nm excitation wavelength); (D) merge of A, B, C images showing no emission in CTRL cells.
